# Analytical techniques for determination of heavy metal migration from different types of locally made plastic food packaging materials using ICP‐MS

**DOI:** 10.1002/fsn3.3391

**Published:** 2023-05-11

**Authors:** Van‐Trong Nguyen, Truong Thi Truc Linh, The‐Ky Vo, Quoc Hung Nguyen, Thanh‐Khue Van

**Affiliations:** ^1^ Faculty of Chemical Engineering Industrial University of Ho Chi Minh City Ho Chi Minh City Vietnam; ^2^ Center of Analytical Services and Experimentation HCMC Ho Chi Minh City Vietnam

**Keywords:** heavy metal, ICP‐MS, migration, plastic food packaging, recovery percentages

## Abstract

Plastic food packaging is an essential element for customer convenience and the preservation of food quality. Nonetheless, heavy metals in the packaging materials, either intentionally or nonintentionally added, can be transferred to the food. Therefore, determining heavy metal contents in these packaging materials is essential. In this study, heavy metals, including Co, Ge, As, Cd, Sb, Pb, Al, and Zn from different intrinsic plastic food packaging materials were analyzed using the inductively coupled plasma‐mass spectrometry (ICP‐MS) method. Moreover, the migration of these elements into the environment was also investigated. This method is validated following the new technique's requirements, which include linearity range, accuracy, precision, the limit of detection (LOD), and the limit of quantitation (LOQ). The method has been suitably validated with the regression equation from the standards prepared in HNO_3_ 1% v/v. The linear range was found to be ~1–20 ng mL^−1^ for Co, Ge, As, Cd, Sb, and Pb and 5–80 ng mL^−1^ for Al and Zn elements. The LODs are ~0.10, 0.25, 0.12, 0.13, 0.11, 0.12, 0.61, and 0.85 ng mL^−1^, and the LOQs are 0.33, 0.83, 0.40, 0.43, 0.36, 0.40, 2.01, and 2.81 ng mL^−1^ obtained for Co, Ge, As, Cd, Sb, Pb, Al, and Zn, respectively. In addition, the recovery percentages received ranged 85.4%–94.1% for Co, 82.6%–95.1% for Ge, 86.3%–97.9% for As, 87.3%–96.3% for Cd, 88.0%–104.4% for Sb, 96.3%–106.0% for Pb, 88.4%–104.0% for Al, and 95.1%–99.7% for Zn. Finally, the migration of these heavy metals from polypropylene (PP) and polystyrene (PS) into foodstuffs was also simulated according to EU legislation, showing that the most leached element was Zn, followed by Al and Pd, with the migration of ~8.38% and ~0.41%, and ~0.19%, respectively.

## Graphical abstract

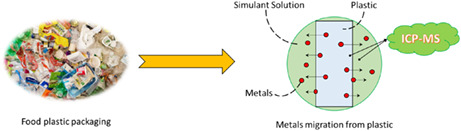

## 1 INTRODUCTION

Plastics are synthetic materials of high molecular weights that can be shaped using a combination of heat, time, and pressure. Polymers are built from small organic molecules, monomers, usually hydrocarbons but often other materials, and are often used in synthesizing plastics. These monomers generally originated from oil or natural gas (Selke et al., 2021). Plastic polymers associated with packaging materials and the packaging industry include polyethylene terephthalate (PET), polypropylene (PP), polystyrene (PS), polyvinyl chloride (PVC), and polyamide (PA). Plastic materials are remarkably resourceful and are used in various applications but dominate in packaging. The packaging plastic for food is made from plastic polymers to which additives are added to attain specific desired properties for an actual application (Whitt et al., 2013). Trace metal contamination of packaging material is a serious problem because the metals might migrate into the food product during storage or be a disposal hazard (Whitt et al., 2016). Metals were added to improve the plastic features with light stabilizers, polymers, or flame retarding agents (Duh, 2002). These additives are not bounded chemically to the matrix of polymeric materials and leach out under the influence of several physicochemical factors such as temperature, solvents, and pH of the stored commodity, leading to the release of toxic substances into air, water, food, food simulants, saliva, sweat, etc. (Mao et al., 2020). Although heavy metal exposure results in numerous adverse health effects, exposure and ingestion of these metals continue dramatically increasing in many countries. A recent study found significant levels of lead, chromium, nickel, antimony, and cadmium in recycled polyethylene terephthalate used for food packaging though the content of Pb in the materials was minor (Whitt et al., 2013). These heavy metals have the potential to migrate on or into food products. All have the potential to cause serious health effects with prolonged exposure or ingestion; all are considered carcinogens by the Agency for Toxic Substances and Disease Registration. Furthermore, long‐term oral exposure and excessive consumption of heavy metals in foodstuffs can lead to diseases such as birth defects, lung, skin, liver, bladder, and kidney cancer, gastrointestinal damage, and even death developed by arsenic (Chowdhury, 2020); Alzheimer's disease and breast cancer by aluminum (Klotz et al., 2017); bone demineralization, severe pulmonary, and gastrointestinal irritant by cadmium (Bernard, 2008); reduced reproductive, taste disorder, and hyposmia by zinc (Reddy et al., 2007); lung disease, heart problems, severe vomiting, stomach ulcers, and diarrhea by antimony (Fujihara & Nishimoto, 2020); gastrointestinal effects (nausea, vomiting, and diarrhea), liver damage, and allergic dermatitis by cobalt (Barreto et al., 2020); acute renal failure, tumor formation, and cancer by germanium (Jinhui & Kui, 1995); kidneys, reproductive system, brain, cause harmful effects on cerebral functioning, infertility, miscarriage, and hypertension by lead (Zounr et al., 2018). The determination of heavy metals in food packaging materials, especially plastics, presents various challenges due to the complexity of the matrix and the extremely low levels of concentration in which the elements are found. Several analytical methods have been developed for determining heavy metals in food packaging samples. Analytical procedures for monitoring concentrations of such elements in packaging materials are usually based on tests involving the migration of elements from the material into a contact solution and then analysis of the extracted metals. A study by Perring et al. (2001) using inductively coupled plasma‐mass spectrometry (ICP‐MS) and inductively coupled plasma‐atomic emission spectrometry (ICP‐AES) identified and quantified the presence of lead, chromium, and cadmium in recycled polyethylene terephthalate food packaging. These metals have the potential to migrate onto and into food if not separated by a functional barrier under normal packaging conditions.

This study aims to validate and apply an analytical method appropriate for determining total cobalt, germanium, arsenic, cadmium, antimony, lead, aluminum, and zinc in various packaging materials widely used in the food industry. Research results provided a reliable procedure to analyze heavy metal traces in widely used plastic materials and warn consumers about plastic food packaging.

## 2 EXPERIMENTAL PROCEDURES

### 2.1 Materials and reagents

Standard solutions (1000 μg mL^−1^) of Co, Ge, As, Cd, Sb, Pb, Al, and Zn and internal standard solutions of Sc, Rh, In, Te, and Ir were purchased from Sigma‐Aldrich Co. Ltd. Nitric acid (HNO_3_ 65%), citric acid (C_6_H_8_O_7_ 99.5%), hydrochloric acid (HCl 37%), and acetic acid (C_2_H_4_O_2_ 99.7%) of ultra‐trace analysis grade were purchased from Merck. Before use, all glassware were cleaned using pure water diluted HNO_3_ obtained through a Sartorius Pure Water System.

### 2.2 GC–MS/MS analytical conditions

A Thermo Scientific™ iCAP™ RQ ICP‐MS was used for all measurements. The sample introduction system consisted of a Peltier cooled (3°C), baffled cyclonic spray chamber, PFA nebulizer, and quartz torch with a 2.5 mm i.d. removable quartz injector. The instrument was operated using kinetic energy discrimination (KED) using pure He as the collision gas in the collision/reaction cell (CRC). To automate the sampling process, an Elemental Scientific SC‐4 DX Autosampler was used. Typical ICP‐MS operating parameters are summarized in Table 1

**TABLE 1:** Instrument operating parameters.

Parameter	Value
Forward power	1550 W
Nebulizer gas	Ar 0.9 L min^−1^
Auxiliary gas	Ar 0.8 L min^−1^
Cool gas flow	Ar 14.0 L min^−1^
CRC conditions	He 4.5 mL min^−1^, 3V KED
Sample uptake/wash time	45 s each
Dwell times	0.01 s
Total acquisition time	3 min
Peristaltic pump speed	40 rpm

### 2.3 Preparation of solutions

The internal standard mixture was prepared at 100 ng mL^−1^ level for each component (Sc, Rh, In, Te, and Ir). An online internal standard addition system automatically added internal standards to the instrument during analysis. The standard solutions were prepared with concentrations of 1.0, 2.0, 5.0, 10.0, and 20.0 ng mL^−1^ for Co, Ge, As, Cd, Sb, and Pb; and 5.0, 10.0, 20.0, 60.0, and 80.0 ng mL^−1^ for Al and Zn. All these solutions were prepared from the stock multi‐standard (1000 ng mL^−1^ for each) by dilution using ultrapure nitric acid (1% v/v) as a diluent that was used for the linearity range study of the method in Table 2. For recovery of the studied metals from plastic food packaging, three concentrations of 0.9, 3.0, and 6.0 ng mL^−1^ for Co, Ge, As, Cd, Sb, and Pb; and 15.0, 45.0, and 60.0 ng mL^−1^ for Al and Zn were prepared, respectively, by spiking of the metals in samples that were immersed in acetic acid (4% v/v) solution to get three spiked concentrations of 0.9, 3.0, and 6.0 ng mL^−1^ for Co, Ge, As, Cd, Sb, and Pb; and 15, 45, and 60 ng mL^−1^ for Al, and Zn. Each sample was analyzed three times, and the results were expressed as mean ± SD (SD: standard deviation). The solutions prepared for linearity had concentrations of 0.9, 3.0, and 6.0 ng mL^−1^ for Co, Ge, As, Cd, Sb, and Pb and 15.0, 45.0, and 60.0 ng mL^−1^ for Al and Zn used for the precision study. To determine the LOD and LOQ of the method, the solution with a concentration of 1 ng mL^−1^ for Co, Ge, As, Cd, Sb, and Pb and 5 ng mL^−1^ for Al and Zn was prepared for linearity study.

**TABLE 2:** Analyte, mass, internal standard, and linear range.

Element	Mass	Internal standard	Linearity range (ng mL^−1^)
^59^Co	58.93	^45^Sc	1.0–20.0
^73^Ge	72.92	^103^Rh	1.0–20.0
^75^As	74.92	^103^Rh	1.0–20.0
^111^Cd	111.90	^115^In	1.0–20.0
^121^Sb	120.90	^121^Te	1.0–20.0
^208^Pb	207.98	^192^Ir	1.0–20.0
^27^Al	26.98	^45^Sc	5.0–80.0
^65^Zn	65.93	^45^Sc	5.0–80.0

### 2.4 Sample preparation

Four different types of plastic food packages, including polypropylene (PP), polyamide (PA), polyvinyl chloride (PVC), and polystyrene (PS) were obtained from local food markets.

#### 2.4.1 Migration tests

According to the guidelines given by the current regulation (Conti & Botri, 1997), 10 × 5 cm of each packaging material were rinsed, immersed, and kept in 100 mL of metals free of acetic acid solution (4% v/v) at 60°C for 30 min. At the end of this treatment, the solution was analyzed for heavy metal concentrations.

#### 2.4.2 Dry ashing method

First, 1 g was cut into small pieces and weighed out in a quartz crucible. After adding 5‐mL nitric acid (65%), the crucible was placed on a hot plate and heated until thin white fumes of SO_3_ were generated, after which the crucible was placed in an oven at 450°C until ashing was completed. After ashing, 5 mL of hydrochloric acid (4 M) was added to the residue, and the liquid was allowed to evaporate to dryness in a water bath. Then, 10 mL of 1 mol L^−1^ nitric acid was added, and the mixture was placed on a hot plate until the residue was dissolved. After cooling, the digest solution volume was adjusted to 50 mL.

#### 2.4.3 The plastic food packaging samples for fortification

The samples were prepared under the same conditions in the previous experiments and spiked with 2.5 mL from the 20 ng mL^−1^ intermediate mixed standard solution for Co, Ge, As, Cd, Sb, and Pb and 3.125 mL from the 80 ng mL^−1^ intermediate mixed standard solution for Al and Zn before extraction solution and digestion, to give final spiking concentration levels of 1 and 5 ng mL^−1^. The extraction and digest solution of samples were then filtered, diluted, and transferred into a 50‐mL volumetric flask. The blank samples were carried out similarly but without using samples containing the metals of interest.

## 3 RESULTS AND DISCUSSION

### 3.1 Validation method

The validation parameters included in this study are linearity, accuracy, precision, the limit of detection (LOD), and the limit of quantification (LOQ). The method validation followed the protocol from EURACHEM guidelines (Bertil & Örnemark, 2014).

#### 3.1.1 Linearity and range

The linearity is the ability of a method to elicit test results that are directly proportional to analyte concentration within a given range. Linearity is reported as the variance of the slope of the regression line. The range is the interval between the upper and lower levels of the analyte that has been demonstrated to be determined with precision, accuracy, and linearity using the developed method. The range is usually expressed in the same units as the results obtained by the process. A minimum of five concentration levels and specific minimum specified degrees are required. Acceptance criteria for linearity are that the correlation coefficient (*R* ^2^) is not <.990 for the least squares method of analysis of the line.

To evaluate the linearity of the method, five calibration standards of the metals with concentrations of 1.0, 2.0, 5.0, 10.0, and 20.0 ng mL^−1^ for Co, Ge, As, Cd, Sb, and Pb and 5.0, 10.0, 20.0, 60.0, and 80.0 ng mL^−1^ for Al and Zn were analyzed by ICP‐MS. A plot of the ratio of response (cps) of the metal analyte divided by the response of the internal standards (Table 2) versus the concentration of the metal resulted in the methodological linear ranges of 1.0–20.0 and 5.0–80 ng mL^−1^, respectively, with a correlation coefficient that is better than 0.995 for the metals analyzed in this study (Table 3). These results demonstrated the linearity of this method over the specified range.

**TABLE 3:** Linearity, LOD, and LOQ in extraction solution (S) and product (P) for PP, PA, PVC, and PS container.

Element	Slope	Intercept	*R* ^2^	LOD (ng mL^−1^)		LOQ (ng mL^−1^)	
				S	P	S	P
^59^Co	68,544	−7038.2	.9994	0.10	0.12	0.33	0.40
^73^Ge	1677.7	−188.17	.9999	0.25	0.25	0.83	0.83
^75^As	4264.4	+181.2	.9999	0.12	0.13	0.40	0.43
^111^Cd	15,304	+3101.1	.9999	0.13	0.15	0.43	0.50
^121^Sb	22,220	−2782.6	.9955	0.11	0.12	0.36	0.40
^208^Pb	202,520	+18,585	.9996	0.12	0.13	0.40	0.43
^27^Al	730.71	−664.67	.9988	0.61	0.65	2.01	2.14
^65^Zn	10,749	+66,015	.9983	0.85	0.89	2.81	2.94

#### 3.1.2 LOD and LOQ

Limit of detection is the lowest concentration that can be distinguished from noise but is not necessarily quantified. The LOD of this method was established by analyzing the blank sample that was spiked with a low content of standard (Section 2.4.3), SD of the low content through the entire analytical process, a total of 10 repetitions was carried out. The limit has been studied as three times SD, according to Equation (1):

(1) LOD=3×SD

Limit of quantitation is the lowest concentration of analyte in the sample that can be determined with acceptable precision and accuracy. The LOQ was studied in the same way as LOD being set as 10 times the SD, according to Equation (2):

(2) LOQ=10×SD

To determine these values, sample blank and spiked samples with an expected concentration of 1.0 ng mL^−1^ for Co, Ge, As, Cd, Sb, and Pb and 5 ng mL^−1^ for Al and Zn were analyzed, and the results are summarized in Table 3. The results show that the method is susceptible to determining heavy metals in plastic packaging samples.

#### 3.1.3 Accuracy of the method

The accuracy of an analytical method, also called recovery, is defined as the closeness between the true value of the analyte in the sample and the value obtained by the analytical procedure. It is a parameter that ensures no loss or contamination occurs during the test procedure, contributing to quantifying the compounds. The samples with low metal contents were spiked with three different analyte levels. The samples were subjected to the highest possible steps of the procedure for at least 3 days with two replicates per day. The mean, standard deviation, relative standard deviation (RSD), and recovery (% Rec) were calculated, where *C*_spk_ was the analyte concentration in the sample spiked; *C*_ref_ was the analyte concentration in the unfortified sample; *C*_add_ is the analyte concentration in the added sample. Mathematically, the % Rec is defined as follows:

(3) $$\%\mathrm{Rec}=\frac{C_{\mathrm{spk}}-C_{\mathrm{ref}}}{C_{\mathrm{add}}}\times 100$$

The ICP‐MS method was evaluated by spiking three known concentrations 0.9, 3.0, and 0.6 ng mL^−1^ for Co, Ge, As, Cd, Sb, and Pb and 5.0, 15.0, and 30.0 ng mL^−1^ for Al and Zn of heavy metals into 4% v/v metals free of acetic acid immersed at 60°C for 30 min (as presented in 2.4.1) with four plastic packaging samples (PP, PA, PVC, and PS container). Similarly, packaging materials (1.0 g product in 50 mL of preparation) were spiked in three known concentrations and then digested by the dry ashing method (as presented in Section 2.4.2). The spike recovery rates are 82%–106% achieved for eight heavy metals, as revealed in Table 4. Good recovery rates indicate that the method has attained accuracy without interference.

**TABLE 4:** Spike recovery rates from extraction solution (S) and product (P) for PP, PA, PVC, and PS containers.

Element	Spike value (ng mL^−1^)	PP		PA		PVC		PS	
		S	P	S	P	S	P	S	P
Co (%)	0.9	85.4	85.0	88.5	88.7	87.5	87.7	86.5	86.1
	3.0	94.1	90.1	96.3	91.3	94.7	92.7	94.7	91.7
	6.0	92.9	91.9	90.7	92.7	93.2	93.5	92.2	92.3
Ge (%)	0.9	82.6	85.5	88.7	87.8	84.5	85.6	82.7	85.7
	3.0	93.8	93.3	93.0	93.0	93.5	93.7	93.1	93.1
	6.0	95.1	94.0	95.8	94.8	95.8	94.6	96.2	92.2
As (%)	0.9	86.3	86.7	87.3	87.0	86.8	86.9	86.9	87.0
	3.0	97.9	95.7	95.6	94.6	97.5	96.1	99.4	95.7
	6.0	92.1	92.1	91.0	91.7	89.7	90.3	93.2	92.1
Cd (%)	0.9	87.3	88.2	89.7	88.7	87.5	88.4	87.8	89.0
	3.0	96.3	95.1	97.6	96.5	95.5	95.7	96.7	96.1
	6.0	96.0	96.5	96.8	94.5	96.3	96.3	97.0	95.0
Sb (%)	0.9	88.0	89.1	89.0	90.0	88.8	92.5	89.1	89.6
	3.0	104.4	98.4	100.1	99.1	102.3	101.1	104.3	100.3
	6.0	102.9	99.9	101.2	99.5	102.7	99.3	105.0	99.5
Pb (%)	0.9	98.8	99.1	99.5	98.5	98.2	98.2	97.3	98.4
	3.0	106.0	99.5	101.5	99.8	101.1	100.1	102.3	102.1
	6.0	96.3	99.8	97.3	99.9	96.8	99.8	95.3	99.3
Al (%)	15	88.4	89.7	89.5	90.0	89.5	89.8	89.3	90.5
	45	100.4	90.8	100.2	92.1	99.5	93.1	100.0	93.5
	60	89.6	95.6	91.2	95.2	89.9	96.0	91.1	95.1
Zn (%)	15	95.1	94.1	96.1	96.0	95.5	95.7	95.6	95.9
	45	95.7	95.6	95.5	96.1	95.9	95.9	96.4	96.2
	60	99.7	98.5	99.1	99.2	99.4	99.0	99.2	99.1

#### 3.1.4 Repeatability

Repeatability is the closeness of agreement between mutually independent test results obtained with the same method on identical test material in the same laboratory by the same operator using the same equipment within short intervals. It is determined from a minimum of nine determinations covering the specified range of the procedure (for example, three levels, three repetitions each). The mean value, standard deviation (SD), and relative standard deviation (RSD) are calculated by using the following formulas:

(4) $$\bar{X}=\frac{\sum_{i=1}^{n}X_{i}}{n}$$

(5) $$\mathrm{SD}=\sqrt{\frac{\sum_{i=1}^{n}\left(X_{i}-\bar{X}\right)}{n-1}}$$

(6) $$\mathrm{RSD}=\frac{\mathrm{SD}}{\bar{X}}$$

The current method's repeatability for determining eight investigated metals was evaluated by spiking three known concentrations of 0.9, 3.0, and 0.6 ng mL^−1^ for Co, Ge, As, Cd, Sb, and Pb and 5.0, 15.0, and 30.0 ng mL^−1^ for Al and Zn. All samples were immersed into 4% v/v metals free of acetic acid at 60°C for 30 min (Section 2.4.1) with four plastic packaging samples (PP, PA, PVC, and PS container). Similarly, packaging materials (1.0 g product in 50 mL of preparation) were spiked in three known concentrations and then digested by dry ashing method (Section 2.4.2), which is found to be less than 10% for all metals at the three concentration levels (data presented in Table 5). These results confirm that the current methodology for determining the heavy metals in packaging materials is repeatable.

**TABLE 5:** RSD% (*n* = 6) from extraction solution (S) and products (P) for PP, PA, PVC, and PS containers.

Element	Spike value (ng mL^−1^)	PP		PA		PVC		PS	
		S	P	S	P	S	P	S	P
Co (%)	0.9	2.2	2.5	2.3	2.7	2.4	2.5	2.1	2.6
	3.0	1.9	2.8	1.8	1.5	1.5	3.5	2.0	2.2
	6.0	0.7	1.6	0.8	1.8	0.9	1.9	0.8	2.8
Ge (%)	0.9	5.4	3.4	5.3	5.3	5.0	3.0	5.1	4.1
	3.0	2.1	2.1	2.0	2.0	2.3	2.5	2.2	3.2
	6.0	2.1	2.1	2.1	2.1	2.5	2.5	2.3	2.5
As (%)	0.9	5.4	4.4	5.5	5.5	5.6	4.6	5.1	4.4
	3.0	2.1	3.5	2.2	2.2	2.4	3.5	2.3	2.5
	6.0	2.1	3.3	2.0	2.0	2.5	3.2	2.0	2.7
Cd (%)	0.9	2.2	2.8	2.1	3.1	2.6	1.6	2.5	2.3
	3.0	1.7	1.5	1.6	2.6	1.9	2.9	1.9	1.2
	6.0	2.0	2.3	2.5	2.8	2.2	3.2	2.5	2.9
Sb (%)	0.9	8.0	3.2	8.3	4.0	7.8	3.6	8.1	4.0
	3.0	5.2	3.5	5.0	2.3	5.0	4.2	5.5	5.1
	6.0	2.4	3.1	2.7	3.5	2.5	3.3	2.2	3.8
Pb (%)	0.9	3.0	3.1	3.1	2.1	3.3	3.0	3.1	4.1
	3.0	3.3	3.4	3.5	3.3	3.7	3.5	3.2	2.2
	6.0	3.5	3.2	3.7	3.1	3.3	3.8	3.5	3.0
Al (%)	15	1.9	2.5	1.6	2.8	1.7	1.5	1.8	2.3
	45	2.8	2.3	2.7	3.2	2.6	2.2	2.7	3.2
	60	0.9	1.4	0.8	2.3	0.9	0.7	0.7	1.9
Zn (%)	15	9.5	6.5	9.0	5.0	8.8	7.4	9.2	9.1
	45	6.8	5.8	6.7	7.7	6.5	7.8	6.6	6.8
	60	6.2	4.2	6.0	6.2	6.2	6.6	6.0	7.1

### 3.2 Migration of heavy metal from plastic packaging materials

The heavy metal content in intrinsic plastic packaging and migrated heavy metals from four plastic packaging materials soaked in solution 4% v/v metals free of acetic acid at 60°C for 30 min were determined and are presented in Table 6. Only metals such as lead, aluminum, and zinc were found with the four types of plastic packaging selected for the analysis, while most other metals were not present. The leaching amounts are in ppb ranges but vary broadly for different plastic packages.

**TABLE 6:** Average levels of detectable heavy metals (ng mL^−1^) from extraction solution (S) and products (P) for PP, PA, PVC, and PS containers.

Element	PP		PA		PVC		PS	
	S	P	S	P	S	P	S	P
Co (ng mL^−1^)	n.d.	n.d.	n.d.	n.d.	n.d.	n.d.	n.d.	n.d.
Ge (ng mL^−1^)	n.d.	n.d.	n.d.	n.d.	n.d.	n.d.	n.d.	n.d.
As (ng mL^−1^)	n.d.	n.d.	n.d.	n.d.	n.d.	n.d.	n.d.	n.d.
Cd (ng mL^−1^)	n.d.	n.d.	n.d.	n.d.	n.d.	n.d.	n.d.	n.d.
Sb (ng mL^−1^)	n.d.	n.d.	n.d.	87.44	n.d.	n.d.	n.d.	412.92
Pb (ng mL^−1^)	n.d.	129.00	n.d.	254.82	0.50	269.8	n.d.	583.88
Al (ng mL^−1^)	3.41	831.25	1.79	2708.35	5.40	1615.5	4.14	15,076
Zn (ng mL^−1^)	53.66	640.31	14.88	5855.9	198.1	3566.7	38.44	4353.1

Abbreviation: n.d., not detected.

**FIGURE 1:**
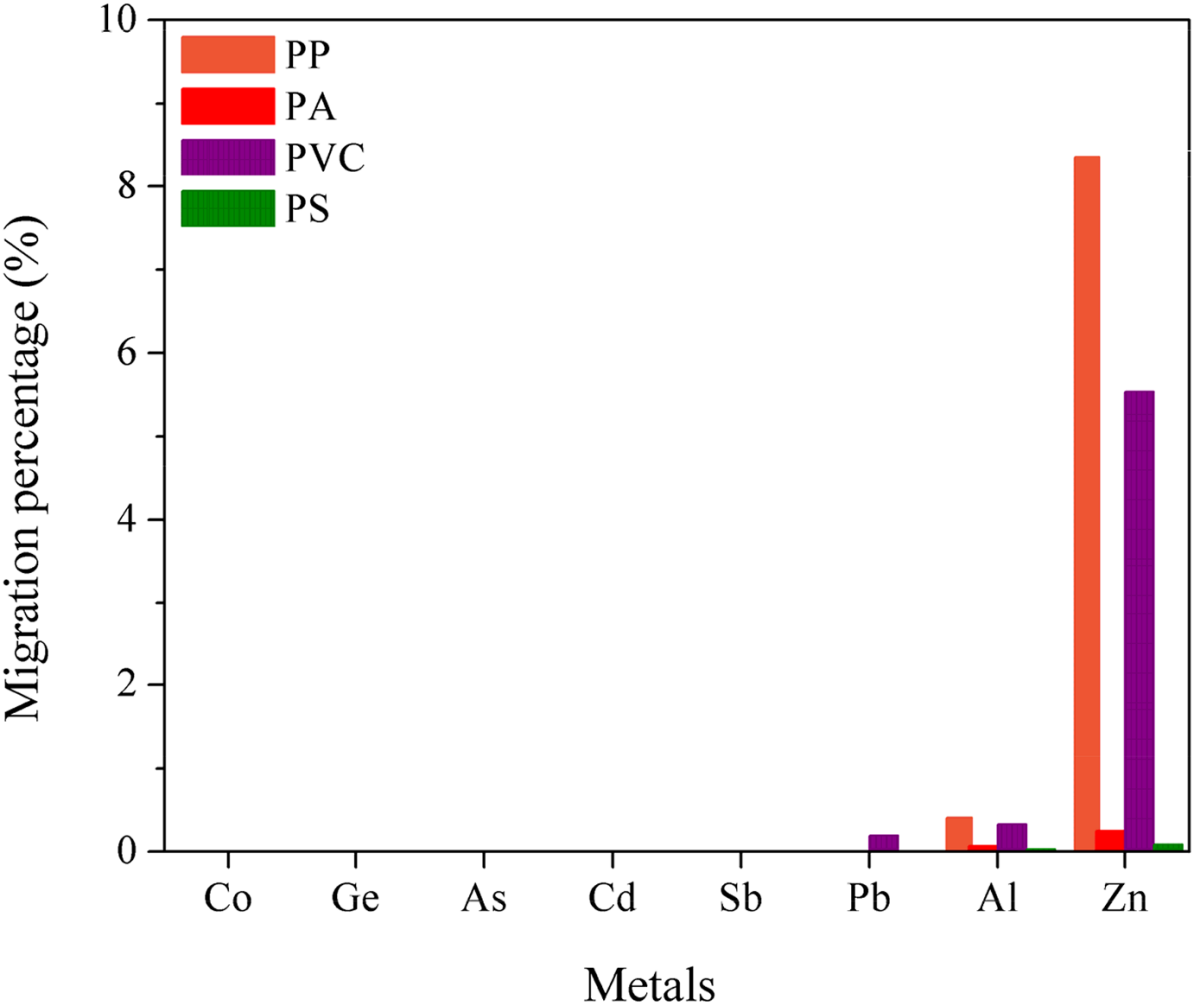
Migration percentage of heavy metals from plastic food packaging materials.

Figure 1 shows that the most leached element is zinc, followed by aluminum and lead, with migration percentages of 8.38% and 0.41%, and 0.19%, respectively, while cobalt, germanium, arsenic, cadmium, and antimony were not detected for any plastic materials. The measured concentrations shown in Table 4 are much lower than the restriction levels for Co, Ge, As, Cd, Sb, Pd, Al, and Zn, as described in EU No 10/2011 (Khan & Khan, 2022).

## 4 CONCLUSIONS

The method has been developed to determine heavy metals in intrinsic plastic food packaging materials and those migrated from them. Optimization and validation of the ICP‐MS method were carried out. It resulted in certifying the analytical parameters such as linearity, accuracy, precision, the limit of LOD, and LOQ of Co, Ge, As, Cd, Sb, Pd, Al, and Zn analytes in plastic food packaging materials. Sample preparation was performed by the dry ashing method. Heavy metal elements migrated from the materials tested by soaking these samples in a 100 mL 4% v/v metals free of acetic acid at 60°C for 30 min. The detected leaching amounts of eight metallic elements are lower than the restriction levels defined by EU No. 10/2011 but varied largely from different plastics. These methods are proper for monitoring the packaging materials used for food products. Therefore, it needs to safeguard consumers' health through awareness of society about the harmful effects of plastic food containers, especially locally made containers with no additives specification.

## AUTHOR CONTRIBUTIONS

**Van‐Trong Nguyen:** Funding acquisition (lead); methodology (equal); writing – original draft (lead). **Truong Thi Truc Linh:** Data curation (equal); resources (equal); validation (equal). **The‐Ky Vo:** Conceptualization (equal); formal analysis (equal); resources (equal); writing – review and editing (equal). **Quoc Hung Nguyen:** Conceptualization (equal); methodology (equal); supervision (equal). **Thanh‐Khue Van:** Project administration (equal); writing – review and editing (equal).

## FUNDING INFORMATION

The authors greatly appreciate the financial support of the Faculty of Chemical Engineering, Industrial University of Ho Chi Minh City (IUH), Vietnam.

## CONFLICT OF INTEREST STATEMENT

The authors declare that they have no conflicts of interest.

## Data Availability

Data sharing is not applicable to this article has no new data were created or analyzed in this study.
